# The harmful effects of partisan polarization on health

**DOI:** 10.1093/pnasnexus/pgac011

**Published:** 2022-03-09

**Authors:** Timothy Fraser, Daniel P Aldrich, Costas Panagopoulos, David Hummel, Daniel Kim

**Affiliations:** Political Science Department, Northeastern University, Boston, MA 02115, USA; Political Science Department, Northeastern University, Boston, MA 02115, USA; Political Science Department, Northeastern University, Boston, MA 02115, USA; Economics Department, Northeastern University, Boston, MA 02115, USA; Bouve College of Health Sciences, Northeastern University, Boston, MA 02115, USA

**Keywords:** health, polarization, politics, partisanship, survey

## Abstract

Partisan polarization significantly drives stress and anxiety among Americans, and recent aggregate-level studies suggest polarization may be shaping their health. This individual-level study uses a new representative dataset of 2,752 US residents surveyed between December 2019 and January 2020, some US residents report more days of poor physical and mental health per month than others. Using negative binomial models, zero inflated models, and visualizations, we find evidence that polarization is linked to declines in physical health: the more distant an individual feels politically from the average voter in their state, the worse health outcomes he or she reports. By uncovering the individual-level political correlates of health, this study aims to encourage further study and attention to the broader consequences of political polarization on American communities.

Significance Statement Recent scholarship indicates that rising political polarization in American communities might have real consequences for individuals’ health. While past studies examined vaccination, mortality, obesity, and health behaviors, most studies focused on the state or county level. Using a representative survey of US residents, this study examines the effect of political polarization on individuals’ overall physical and mental health. Measuring political polarization from 0 to 10, we found strong links with health. Compared to individuals quite similar to their state's average voter (0), respondents with strongly diverging political views (10) experienced 2.07 more days of poor physical health per month, or an extra 6.9% chance every day.

## Introduction

Political polarization has grown precipitously since the 1970s, especially among activists (1, 2), culminating in what some scholars have dubbed “fear and loathing” across party lines (3). Recent scholarship has shown this wave of polarization can have potent consequences, including on individuals’ health (4, 5). For instance, aggregate-level studies have linked county-level presidential election voting preferences to changing health outcomes, including mortality and vaccination rates and obesity (6–10). In the current study, we explore further whether partisan polarization is linked to health outcomes. Specifically, we examine whether individuals who feel more politically polarized and distant from their communities report worse health outcomes.

Our study analyzes original survey data collected in December 2019 and January 2020 to examine the relationship between respondents’ perceptions of the levels of political polarization in the communities in which they are embedded and their physical and mental health. As a preview, we find that individuals who consider their environments to be polarized report worse physical health outcomes. We find strong evidence that political polarization, compared to the median voters in their communities, matters most at the state level, with some descriptive evidence that this trend may apply at the national level too.

This study makes 4 main contributions. First, our findings demonstrate at the individual level that political polarization exerts strong associations with poor health (4, 5, 7, 10), and that these associations are *independent* of party affiliation. Second, while a broad range of factors contribute to health, we observe significant associations between polarization and health outcomes, even after controlling for a robust set of socio-demographic and health-related covariates including health conditions, race, gender, age, education, and partisanship. Third, we find that polarization at different levels of government correlates with health differently: greater ideological distance from the median voter in one's state is linked to worse physical health, while greater distance from the median US voter is mostly unrelated. Finally, we find that the political polarization asserts even more deleterious effects for those with especially poor health, implying that vulnerability and polarization are deeply, problematically related. Though no one panacea will cure these divides, these insights will help public health officials, decision-makers, and scholars to understand the distinct drivers of well-being in order to tailor policies to address specific issues.

## Background and Expectations

This study examines whether (and how) political polarization affects health among Americans. Below we summarize key highlights from the extensive literature that examines the determinants of health outcomes in order to account for a wide range of potential confounding factors in our aim to isolate the impact of polarization on health. Specifically, we review how demographics, health conditions, behaviors, health care policy, political partisanship, and political polarization shape health outcomes.

### Health conditions and behaviors

First, some individuals might face worse health outcomes due to the health conditions they face and behaviors they adopt. Tobacco consumption, obesity, poor diet, alcohol and drug use, type II diabetes, and high blood pressure were found to be the top 6 causes of change in American life spans between 1990 and 2016 (11). Smoking, in particular, is a high priority by the Centers for Disease Control and Prevention, because smoking causes more deaths each year than HIV, illegal drug use, alcohol use, motor vehicle injuries, and firearm injuries combined (12).

### Policy determinants of health

Alternatively, health outcomes might vary among individuals due to policy changes that affect health habits, environmental exposure, and stressors. For example, the geographic distribution of health care facilities and access to affordable fresh produce limit access to health care services and predisposes communities to obesity (13–15). Similarly, communities with better quality hospital care, commonly depicted through lower hospital readmission rates, tend to see lower mortality rates (16). However, macro-level government policies also shape these health outcomes. Immunization rates, efforts to control smog pollution and exposure to particulate matter (PM 2.5), health insurance rates due to government enrollment efforts, the extent of unemployment benefits, higher welfare spending, and income assistance programs like Earned Income Tax Credits have all been linked to better health outcomes (16, 17–19, 20–22).

Further, certain party platforms have differing implications for health outcomes. In the late 2010s,  scholars noticed that states with governors and legislatures run by Democrats rather than Republicans were more likely to endorse and adopt nutrition and physical activity policies and CDC community intervention strategies for obesity, rather than personal responsibility approaches, in neighborhoods, parks, schools, and workplaces (23, 24). Partisanship also closely shapes health insurance, a key factor in access to healthcare: today, one relevant health insurance policy affecting Americans nationwide is the Affordable Care Act; state-level and national-level efforts to undo or hinder it through legal challenges have created a patchwork quilt of insurance coverage across the country (25, 26). Residents who are uninsured face much greater financial challenges when seeking health care, leading to long-term declines in physical or mental health (27).

### Demographic vulnerability

Demographics also correlate with individuals’ health outcomes. Communities of color, particularly Black communities, face dramatically lower life expectancy than predominantly white communities, due to systemic racism (28). Racism shapes health outcomes in through race-related discrimination in work and health care access, the health and economic impacts of racial profiling and incarceration, environmental exposure to stress and pollutants, and long-term inequalities in education and poverty (20, 29–31). Likewise, age and gender shape health as well; areas with higher concentrations of elderly tend to experience greater mortality rates, while women tend to have greater life expectancy and disease prevalence than men (20). Finally, socioeconomic status (SES), both in terms of income and education, also shapes health outcomes, via poverty, education, and SES-related stress. An analysis of the American Association of Retired Persons (AARP) members from 1,925 to 1,945 showed that educational attainment dramatically boosts overall health (32), while increases in long-term unemployment are associated with increased mortality, due to the stress of covering expenses for family when unemployed (33).

### Political partisanship

Studies also reveal that individuals’ political views may influence their health. There is evidence of considerable partisan asymmetries in certain health-related behaviors. For example, individual level surveys show that Republicans and conservatives tend to consume fewer fruits and vegetables and more fat and processed foods, exercise less, get flu vaccines less, and search for health information less, but also drink and smoke less often compared to Democrats and liberals, for example (34). Similarly, a study of Medicare Part D recipients found that residents in counties that voted for Trump in 2016 were more likely to receive prolonged opioid prescriptions than the average county (9).

On the other hand, political partisanship can shape health en masse, by shaping rates of societal and community adoption of protective behaviors, like vaccination. For example, adolescents in states that voted Democratic in the 2012 presidential election were much more likely to have received vaccines for human papillomavirus (HPV), tetanus–diphtheria–acellular–pertussis (Tdap), and meningococcal conjugate (MCV4) than adolescents in states that voted Republican (10).

Scholars have also linked health to political outcomes. Counties suffering higher levels of deaths of despair, below-average gains in life expectancy, and disease prevalence, for instance, were more likely to vote for Donald Trump in 2016 (7, 8, 35–37). Similarly, Smith and colleagues (38) found that, “Democrats, self-identified liberals, those who are socially and economically liberal, and people who disapprove of President Donald Trump are, across the board, more likely to report negative health impacts from politics”.

### Political polarization

Fewer studies have examined how political polarization specifically affects health. Such a link is theoretically conceivable given that polarization in communities can cause stress and anxiety, causing physical and mental health to deteriorate. Some scholars have argued that Americans’ political and ideological views have shifted considerably toward extremes since the 1990s (1, 39–41). While other researchers reject this perspective (2, 42–45), studies have revealed clear changes in how Americans view members of the opposing political party (3, 46, 47). Negative advertising and increased exposure to campaigns has reinforced voters’ partisan identities and beliefs about the opposing party (46). Anecdotal evidence suggests that voters and elites perceive their communities to be increasingly polarized, whether or not they are, and some attribute this to echo chambers in social media (48, 49). Other studies disagree, showing that social media actually results in greater exchange of cross-cutting political views (50–52), and that ordinary social media users tend to share mainstream content, while political elites are responsible for sharing more partisan content (53). Social media enables voters to learn about the political views of distant friends and acquaintances, which they may not have known otherwise, leading them to feel that partisan polarization is rising (54, 55).

Recent studies suggest also that partisan citizens discriminate against partisan others, even more so than against people of different races, leading citizens to consider politics increasingly important in selecting their spouses and facilitating echo chambers in the home (3). Strong partisans with extreme views are especially likely to perceive polarization occurring, viewing partisan others as caricatures (56), especially when provoked to anger, which motivates people to adopt political information biased toward their side (57). Partisan media outlets capitalize on this, accelerating political information sharing by fostering anger from audiences (58); political disinformation on Facebook appears to exacerbate this, fueling anger and incivility more so than real political news (59). Experimental research suggests a relationship between individuals’ partisan polarization and degree of intergroup anxiety, where less openness to participating in political discussions is associated with greater anxiety (60). A 2017 report issued by the American Psychological Association warned that politics is a significant source of stress for the average American (61). And while many Americans are not equally informed about politics (62), we anticipate residents may still be affected by day-to-day experiences of political polarization, through reduced interactions with members of the other party, a hesitancy to discuss sensitive topics with others, and loss of trust in neighbors, friends, or family,

In light of these findings, we hypothesize that political polarization negatively impacts individuals’ overall wellbeing (7, 10, 36). We argue that polarization affects health, above and beyond the impact of health conditions and behaviors, health policy, demographic vulnerability, or politically partisanship and partisan health behaviors. Below, we introduce our data and methods to test the relationship between polarization and health.

## Methods

**Fig. 1.**
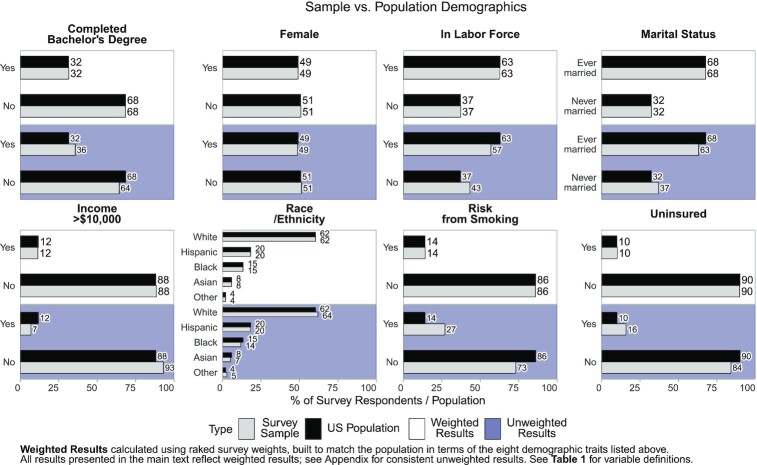
Sample vs. population demographics.

This study examines why some individuals experience worse health than others, and to what degree political polarization is associated with their health outcomes. To examine this, we partnered with Qualtrics to conduct a national survey of English-speaking US residents. The survey, conducted between 2019 December 23 and 2020 January 3, yielded 2,752 completed responses and used quota sampling procedures to produce a sample designed to be representative of the US population. Figure 1 demonstrates that our original, unweighted sample is quite similar to the population in terms of gender, race, income, marital status, bachelor's degrees, unemployment, rates of smoking, and health insurance coverage. To account for slight differences in rates of smoking, poverty, and insurance, we weighted the survey based on these 8 demographic categories above (see SI Supplementary Text). As a result, our weighted results in Fig. 1 match the population almost exactly.

All methods in this survey were carried out in accordance with relevant guidelines and regulations; our research protocol was approved by the Institutional Review Board at Northeastern University (protocol # 18–11–14). Finally, informed consent was obtained from all respondents, who were all over age 18. Below, we summarize the variables from this survey used in our analyses and describe our modeling techniques.

### Outcomes

As our main outcome variables, we examine physical and mental health using 2 self-reported measures. For physical health, we asked respondents to tell us how many days in the past month they experienced poor physical health. We followed a similar approach for mental health, asking respondents how many days in the past month they had experienced poor mental health. These questions measuring self-reported overall health are commonly employed in the Behavioral Risk Factor Surveillance System, the largest survey of health conditions in America, using their most recent wording. These variables range from 0 to 30 days. (For details, see Figure S1 (Supplementary Material) in this article's Supplementary Information (SI)). Further, they demonstrate strong predictive validity (see Figure S3, Supplementary Material) and conceptual validity (see Figure S4, Supplementary Material) when compared to other related measures.

### Independent variables and covariates

We operationalize polarization, our main independent variable of interest, as follows. We measure respondents’ *perceived polarization* using several benchmarks for comparison. Respondents were asked to rank themselves, the average voter in their state, and the average voter in the United States in terms of their political views, using a feeling thermometer on a scale from 0 to 10, where 0 equals most liberal, 10 equals most conservative, and 5 equals neither liberal nor conservative. Then, we calculated the absolute difference between each respondent and the average voter in their state, which we refer to as *state-level perceived polarization*, as well as the absolute difference between each respondent and the average voter in the United States, which we refer to as *national-level perceived polarization*. A value of 0 means that individuals rate themselves as politically identical to the average voter in their state or country, while a value of 10 indicates that they rate their political views as extremely far from the average voter.

Past studies measured polarization by asking how upset respondents would be if their child married a member of the opposing party (63), candidates they would never vote for (64), and gauging respondents’ implicit bias towards parties (3). In contrast, this measure adds to the literature by capturing how politically isolated residents feel relative to their (a) *state* and (b) *country* at large. This helps us measure both the intensity of people's political views towards the opposing party, as past polarization studies have done (3, 46), but also measure the process of separation and isolation by which polarization might viably affect health. This measure has been applied by several recent studies to evaluate perceived polarization (4, 5).

Further, our analyses control for a wide range of additional variables. For partisanship, we asked respondents the American National Election Survey's 7-point partisan identification scale, identifying each respondent as a (1) Strong Democrat, (2) Weak Democrat, (3) Leaning Democrat, (4) Independent, (5) Leaning Republican, (6) Weak Republican, or (7) Strong Republican. To adjust for the strength of partisanship, we then collapsed our 7-pointt partisan identification scale into a 4-pointt scale, from 0 to 3. We classified independents as (0) *nonpartisans*, Leaning Democrats or Republicans as (1) *leaning partisans*, Weak Democrats, or Republicans as (2) *weak partisans*, and Strong Democrats or Republicans as (3) *strong partisans*. To control for pre-existing health conditions that might otherwise affect health, we used respondents’ body-mass index (BMI), calculated from their self-reported weight and height. We also adjusted for residents at risk from smoking, defined by the BRFSS as respondents who smoke at least 100 cigarettes in their lifetimes and currently smoke some days or every day. Finally, to adjust for health policy, we asked respondents whether they had access to any form of health insurance or not.

We also account for a series of additional, standard demographic controls including age, race, gender, educational attainment, income, employment, marital status, and religion (see Table 1 for a summary of measures and Tables S1 and S2 (Supplementary Material) for details and descriptive statistics).

**Table 1.** Definition of variables.

Type	Concept	Measure	Level of measurement
**Outcome**	**Poor physical health**	Self-reported days of poor physical health per month	Discrete (0–30)
	**Poor mental health**	Self-reported days of poor mental health per month	
**Independent variables**	**Mass political polarization**	Perceived difference between self vs. average state voter	Continuous (0–10) (0 = lowest, 10 = greatest)
		Perceived difference between self. vs. average US voter	
**Covariates**	**Partisanship**	Party identification	Ordinal (1–7) (1 = Strong Dem; 7 = Strong Rep.)
		Strength of partisanship	Ordinal (0–3) (0 = nonpartisan; 1 = weak partisan; 2 = moderate partisan; 3 = strong partisan)
	**Health conditions**	BMI ^1^	Continuous
		At risk from smoking ^2^	Binary (yes/no)
	**Health insurance**	% uninsured	Binary (yes/no)
	**Basic demographics**	Age	Continuous
		Gender	Binary (woman/other)
		Income ^3^	Ordinal ranking from 1 to 11 (lowest to highest)
		Race/ethnicity	Categorical (Black, White, Asian, Hispanic, and other race)
		(%) Some college or more	Binary (yes/no)
	**Extended demographics**	Employment status	Categorical (employed, unemployed, not in labor force)
		Marital status ^4^	Binary (never married: yes/no, where no = married, separated, widowed, or divorced)
		Religion	Categorical (Protestant, Catholic, other Christian, Jewish, Muslim, other religion, and no religion)
**Fixed effects**	**State**	State	50 US states

1 BMI was calculated after surveying respondents’ self-reported height and weight.

2 Risk from smoking: we adhere to the BRFSS definition for risk from smoking, defined as a person (1) having smoked at least 100 cigarettes in their lifetime and (2) currently smoking every day or some days.

3 Income was simplified into an 11-point scale, where 1 = less than $10,00, 2 = $10,000–$19,999, 3 = $20,000–$29,999, 4 = $30,000–$49,999, 5 = $50,000–$69,999, 6 = $70,000–$99,999, 7 = $100,000–$124,999, 8 = $125,000–$149,999, 9 = $150,000–$199,999, 10 = $200,000–$249,999, and 11 = $250,000 or more.

4 Marital status was simplified into 2 categories: “never married” (36.9% in our sample vs. 32.3% in the population), and “other” (63.1% in our sample, vs. 67.7% in the population). Raked weighting required us to simplify categories due to the very small percentages of widowed (9∼11%), divorced (∼5%), or separated individuals (∼2%), relative to other categories.

## Estimation and Statistical Procedures

To model our 2 outcomes of interest (days of poor physical health and days of poor mental health per month), we estimate negative binomial models which best capture the right skewed nature of our positive skewed, over-dispersed outcome variables. (The skewed nature of our key outcome variables is visually depicted in Figure S1, Supplementary Material). Negative binomial models fit better than Poisson models, and, although our data is strongly zero-inflated, our estimates reveal substantively similar results when using either negative binomial models (Tables S3 and S4, Supplementary Material) or zero-inflated negative binomial models (Tables S5–S8, Supplementary Material). Zero-inflated models are 2-component mixture models which treat the data as if there is one process generating the count portion, including some zeros, and another generating the point mass at zero. This method produces 1 set of effects for 1 or more days of poor health (Tables S5 and S6, Supplementary Material), and another set of effects predicting the likelihood of a response of 0 days vs. 1 day of poor health (Tables S7 and S8, Supplementary Material). Since results are quite comparable, we report results from negative binomial models, which are easier to interpret and more familiar to readers. In both sets of models, we controlled for state-by-state differences using fixed effects for each state, to account for state-specific policy interventions and how differences in state size and geography might shape polarization and health. All models were conducted using raked survey weights; unweighted results were also consistent and are shown in Tables S4, S6, and S8 (Supplementary Material). Results for our independent variable remained consistent even without controls for BMI, smoking, and state effects (see Table S9, Supplementary Material), or for strength of partisanship and state effects (see Table S10, Supplementary Material). This indicates our results are not artifacts of any 1 model specification.

Before moving to the presentation of the results, we note that some variables were missing data. For instance, 14% of individuals did not list their income bracket, 1.8% of respondents were missing partisan affiliation, and 4.5% were missing BMI indicators like height or weight. Typically, scholars avoid using variables missing more than 5% of data points, but these controls are central to this study. Multiple imputation is the gold standard for dealing with such situations because it imputes missing data points by drawing from latent trends in the data and has shown to be robust even with high levels of missing data, even greater than 20% (65). Fortunately, our main findings are consistent both when omitting cases with missing data, when imputing them with the means, or when using multiple imputation. (We present the results of models using multiple imputation (66), the most robust method, in Tables S3–S8, Supplementary Material).

Our models explain approximately 11%–16% of the variation in physical health and mental health, based on their Nagelkerke R-squared value. This is expected, as many other environmental, behavioral, and genetic factors that shape health that are not captured in our models. Our models also revealed no problematic multicollinearity; in fact, all variables across models showed variance inflation factor scores below the threshold of 2.5, well below problematic scores of 10 or higher. Below, we report these results and visualize findings. All *P*-values reported reflect 2-tailed tests.

## Results

This study aimed to test why some Americans experience worse health outcomes than others, testing the association of political polarization with health outcomes from a survey of 2,752 respondents. We report the estimated effects of state- and national-level perceived polarization and other covariates on each outcome in Tables S3–S8 (Supplementary Material). Since the results of our zero-inflated models were nearly identical to a simple negative binomial model, we report results from the latter below.

### Statistical model results

First, we found a statistically significant relationship between state-level perceived polarization and the number of days of poor physical health per month that a respondent reports. Based on the incidence rate ratio (irr) of polarization, for each 1 unit increase in state level perceived polarization on a scale from 0 to 10, the chances of an extra day of poor physical health grow by 1.03 times, all else constant (*P* = 0.019). Our estimates also imply that individuals are 1.02 times as likely to experience an extra day of poor physical health as perceptions of national level polarization increase (*P* = 0.178), but this association was not statistically significant at conventional levels. (For our zero-inflated models, this association was somewhat significant at *P* = 0.09). Finally, state and national level polarization were slightly related to mental health outcomes (irr = 1.02, *P* = 0.330 and irr = 1.01, *P* = 0.561, respectively), but these associations were not statistically significant.

How meaningful are these results? For context, our mean respondent reported 6.05 days of poor physical health out of a month (30 days), meaning the average American has a 20.2% chance of poor physical health *daily*. Further, our mean respondent scored 2.15 on our scale for perceived state-level polarization. Our irr projects that a score of 5 boosts their daily chance of poor physical health to 22.4%, a score of 7 boosts it to 24%, and a score of 10 boosts it to 26.4%. A 6.2% increase in one's chance of poor physical health on a given day, out of 100%, is quite considerable. Overall, we view the preponderance of the empirical evidence to support our expectation that polarization is negatively related to health outcomes, even as some of the relationships we observe are not statistically definitive.

Several, related relationships we observe lead us to have further confidence in our findings. The negative binomial models, for example, revealed several, expected associations. First, respondents' BMI was positively associated with all poor health outcomes (irr = 0.018∼0.023, *P* < 0.001). We also found that uninsured respondents reported more days of poor mental (irr = 0.104∼0.161, *P* = 0.191∼0.347) and physical health (*P* = 0.615), though these associations were not statistically significant at conventional levels. Finally, we also observe a consistent positive uptick in poor health among unemployed respondents (irr = 0.412∼0.585, *P* = 0.002 ∼ 0.054), which matches extant literature that suggests unemployment induces considerable stress and impacts quality of life (20).

### Visualization

**Fig. 2.**
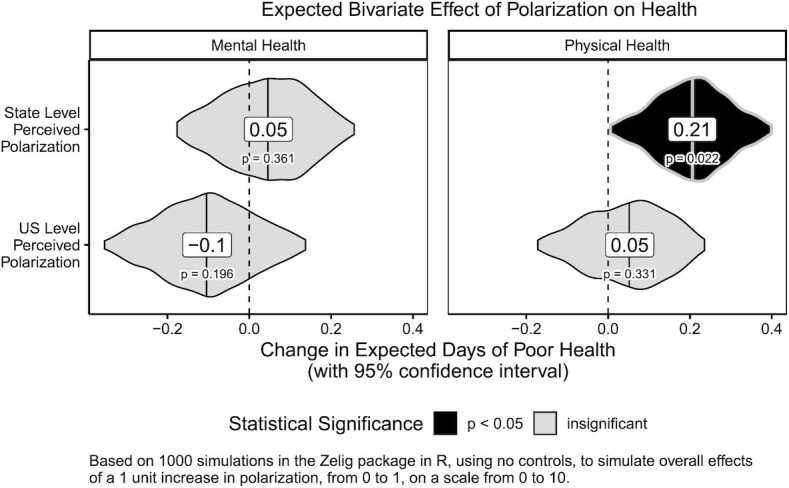
Expected bivariate effect of polarization on health.

For visual representations of our key findings, we visualized the results in 3 ways. First, we simulated the expected association between polarization and poor health from simple bivariate negative binomial models as descriptive statistics, using 1000 simulations in the Zelig package in R (67, 68). This depicts the overall association between polarization and health, before controls, to show that our results are not results of a certain model specification but instead are visible at large in the data. These are visualized in Fig. 2 as violin plots depicting a 95% CI around change in expected outcomes, with lines and labels indicating the median expected change projected as levels of perceived polarization, measured on a scale from 0 to 10, increases by 1 unit from 0 to 1. We see that a 1 unit increase in a respondents’ degree of political polarization is linked to a statistically significant expected increase of 0.21 days of poor physical health, plus or minus 0.19 days (95% CI, *P* = 0.022). These bivariate tests also confirm, like our models, that state-level perceived polarization is more clearly related to declining physical health than national-level perceived polarization.

**Fig. 3.**
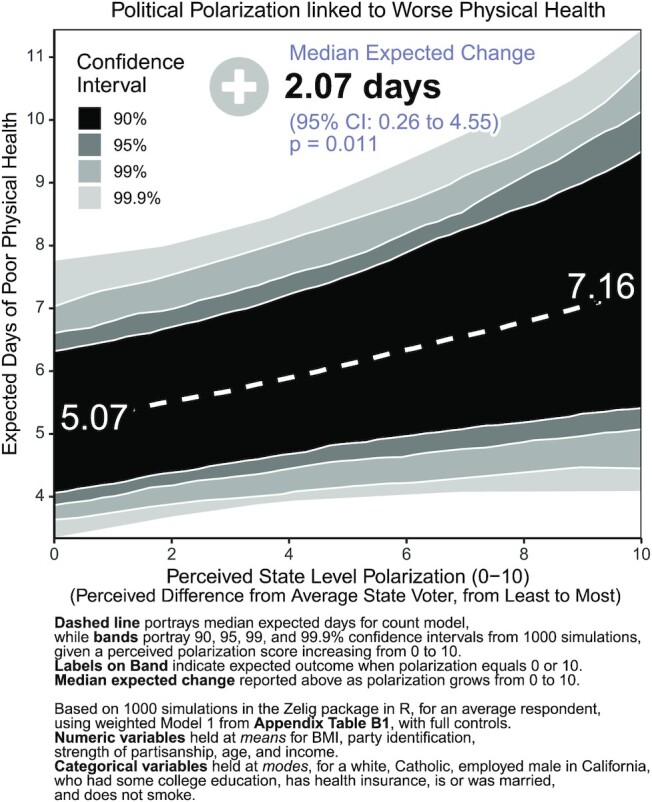
Political polarization linked to worse physical health.

Second, we visualized the association between state-level perceived polarization and poor physical health, controlling for the full battery of available covariates. Here, we simulated the expected change in the number of days of poor physical health as state level polarization increases from 0, representing complete perceived political similarity, to 10, representing maximum perceived political difference, using the Zelig package in R (67), holding all other predictors at their means or modes. Panels show the median expected outcome, alongside CIs of 90%, 95%, 99%, and 99.9% based on 1,000 simulations. The strong association between polarization and days of poor physical health is depicted in Fig. 3. Controlling for all covariates, the impact of polarization persists even after accounting for a wide range of factors, including health conditions, health insurance status, partisanship, or demographic traits. Figure 3 depicts that an average respondent who feels politically similar to the average voter in their state (perceived polarization = 0) experiences 5.07 days of poor physical health, plus or minus 1.36 days (95% CI), but a similar respondent who feels extremely politically different from the average voter in their state (perceived polarization = 10) experiences 7.17 days of poor physical health per month, plus or minus 2.45 days (95% CI). Respondents experienced a median expected change of + 2.07 days (*P* = 0.011) overall.

Third, and for added context, we examined whether the estimated impact of polarization is stronger among individuals who experienced more or fewer days of poor physical health than average. In our sample, the median respondent reported experiencing 2 days of poor physical health per month. We estimate 3 separate models: (1) on all individuals in our sample; (2) on individuals reporting *below* the median number of days of poor physical health; and (3) on all individuals reporting a number of days of poor physical health *above* the median. Each time, to adjust for zero-inflation, we modeled just the count-portion of the outcome, meaning individuals reporting at least 1 day of poor health. Then, using the same approach as in Fig. 3, we simulated from each model the expected number of days of poor physical health per month for an average individual with increasing levels of perceived polarization.^1^

**Fig. 4.**
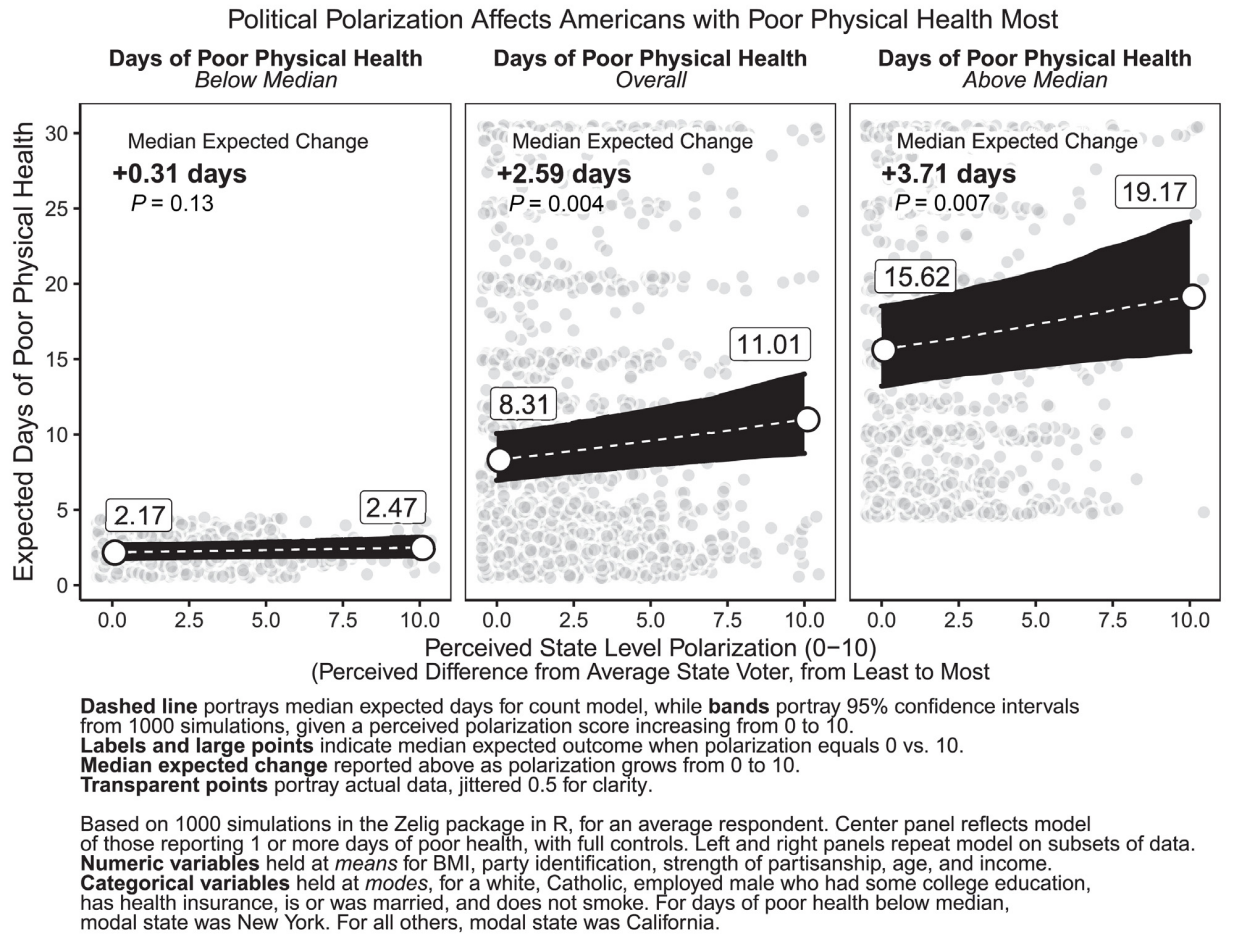
Political polarization affects Americans with poor physical health most.

Figure 4 reveals that polarization's association with poor health is weaker for those with relatively good physical health, but the association grows stronger for those with worse health. For residents who reported 1–2 days of poor physical health per month (left panel), Fig. 4 shows that perceived polarization is associated with a + 0.31 day change (*P* = 0.13) from 2.17 days of poor physical health on average given no perceived polarization (0) to 2.47 days on average given extreme perceived polarization (10). For residents who reported 1–30 days of poor physical health (center panel), Fig. 4 shows that perceived polarization is associated with a + 2.59 day change (*P* = 0.004) from 8.31 days of poor physical health given no perceived polarization (0), to 11.01 days of poor physical health given extreme perceived polarization (10). Finally, for respondents who reported between 2 and 30 days of poor physical health (right panel), Fig. 4 demonstrates that perceived polarization is associated with a + 3.71 day change (*P* = 0.007) from 15.62 days of poor physical health given no perceived polarization (0) to as high as 19.17 days given extreme perceived polarization (10). In other words, when we zoom in on those individuals reporting the worst health (above the median number of days per month), the negative association between state level perceived polarization and health becomes much stronger.

We repeated this analysis for days of poor mental health in Figure S2 (Supplementary Material). Though weaker, state level polarization and mental health follow an opposite trend compared to physical health in Fig. 4. Respondents with below-median levels of poor mental health saw the most significant increase in days of poor mental health when polarization increased from 0 to 10 (+0.74 days, *P* = 0.02). In other words, political polarization may be linked to declines in mental health among those with fewer mental health challenges, but it does not seem to make statistical significant impacts on residents experiencing frequent poor mental health. Perhaps polarization affects different mechanisms for mental vs. physical health, raising anxiety generally among the broader population, but cutting off neighborly support to those with substantial physical health challenges.

## Discussion and Conclusion

This study examines how political polarization associates with physical and mental health. Although some of the findings we report above are mixed or inconclusive, we report several statistically significant results that support the notion that individuals who perceive their environments or communities to be politically polarized report poorer health. Interestingly, our analyses revealed some compelling differences across types of communities. We uncovered consistent patterns that individuals who feel more politically different from the average voter in their state report more days of poor health outcomes, while the relationship for perceived polarization relative to the nation as a whole was more muted. This highlights the importance of deviations from state political views, rather than national political views alone, when considering the contextual impacts of polarization on the population.

We also observed differences across health types, specifically physical and mental health: mental health declines were primarily seen in those with fair mental health, rather than poor mental health (Figure S2, Supplementary Material), whereas physical health declines were seen much more broadly (Fig. 4). We find concerning evidence that polarization's strongest links to poor health occur among vulnerable residents experiencing frequent physical distress, many days a month. Polarization could lead to this outcome by deterring residents from connecting with friends and neighbors, due to diverging political and social views, such that when vulnerable residents need help in the event of illness, injury, pain, or other conditions, they have fewer sources of aid to turn to. These nuanced findings suggest subsequent research is necessary to examine the relationship between polarization and health more thoroughly and to reconsider the theoretical linkages between these concepts.

Our findings have broad implications for policymakers and scholars. First, our results suggest that political polarization is not only problematic for policymaking and governance, it also appears to affect ordinary citizens in very direct ways, including their health. Polarization may not only be a political challenge; it may also be a public health concern. As such, policy responses or community-based interventions designed to reduce, minimize, or counteract extreme polarization may be required, in part to improve citizens’ health.

As social epidemiologists have argued (69, 70), it is vital to focus not just on how public health interventions can benefit high risk individuals, in this case, politically extreme or isolated residents, but how those interventions can alleviate polarization in society broadly (71). After all, one is not polarized alone; stemming polarization requires population and community-level efforts. On top of this, the COVID-19 crisis has deepened political divides in many communities over masking, vaccination, and testing protocols (72). Some scholars have begun developing and testing toolkits for bridging divided communities (73–75). Others highlight techniques for facilitating intergroup contact, citizen assemblies for negotiating neighborhood issues, youth groups (76), as well as investment in community centers (77) and social gathering places (78). These may prove useful for building new ties across party lines. We encourage further study and attention to the broader consequences of political polarization on American communities.

## Supplementary Material

pgac011_Supplemental_File

## Data Availability

Our dataset and replication code are available on the Harvard Dataverse at the following link: https://doi.org/10.7910/DVN/YAOUE1.

## Footnotes

1 Annotation icons in visuals sourced from FontAwesome.
