# Supplementary material for: The harmful effects of partisan polarization on health
Source: PNAS Nexus. 2022 Mar 9;1(1):pgac011. doi: 10.1093/pnasnexus/pgac011 (PMC9802430; doi:10.1093/pnasnexus/pgac011)
Supplement: pgac011_Supplemental_File [file pgac011_supplemental_file.docx]

**Supplementary Information for**

The Harmful Effects of Partisan Polarization on Health

Timothy Fraser*, Daniel P. Aldrich, Costas Panagopoulos, David Hummel, Daniel Kim

Corresponding Author: Timothy Fraser

Email: [timothy.fraser.1@gmail.com](mailto:timothy.fraser.1@gmail.com)

**This PDF file includes:**

Supplementary Text

Figures S1 to S4

Tables S1 to S10

SI References

Our dataset and replication code are available on the Harvard Dataverse at the following link: <https://doi.org/10.7910/DVN/YAOUE1>

**Supplementary Information Text**

### Additional Modeling Details

### Raked Survey Weighting. This survey used a quota sample of 2,572 respondents from a nationally-representative survey of English-speaking US residents, conducted by the survey market research firm Qualtrics in December 2019. We used quotas in terms of gender, age, race, and party to generate a sample of respondents that closely matched the US population. Further, the final samples *also* closely matched the US population in terms of a wide range of relevant demographics. Figure 1 highlights that our sample closely matched the population in terms of each of the following 8 traits: gender, race, income, marital status, education, labor force participation, health insurance coverage, and risk from smoking. (We adhere to the BRFSS definition for risk from smoking, defined as a person (1) having smoked at least 100 cigarettes in their lifetime and (2) currently smoking every day or some days.)

However, some slight differences remained; to account for these differences, we used raked survey weighting, also known as iterative proportional fitting. Raked weighting is the gold standard in the field, used by the US Census, Pew Research Center, and most social surveys as a flexible form of post-survey weighting that can account for differences in numerous demographic variables at the same time.

Survey weights were scored using raked survey weighting using the *survey* package in R (Lumley 2020). The raking process assigned a relative importance score to each respondent based on the eight aforementioned variables from **Figure 1** (gender, race, income, marital status, education, labor force participation, health insurance coverage, and risk from smoking)**.** (Note: For weighting, marital status was simplified into two categories: “never married” (36.9% in our sample vs. 32.3% in the population ), and “other” (63.1% in our sample, vs. 67.7% in the population). Raking required us to simplify categories due to the very small percentages of widowed (9~11%), divorced ( ~5%), or separated individuals (~2%), relative to other categories.) This produces weights ranging from 0.16 to 3.54. Finally, as is good practice, we trimmed weights down to a minimum of 0.3 and a maximum of 3.0, to ensure that no single respondent was up-weighted or down-weighted too much, so as to avoid introducing any undue bias (Potter 1990).

All results and simulations presented in the main text use the weighted models. For comparison, readers can consult the tables below for weighted models, which deliver the same results for our polarization variables of interest.

**Model Validation Exercises.** To further validate our models, we underwent several tests. We added raked survey weights, shown in Tables S3, S5, and S7. Results remained consistent with unweighted models, shown in Tables S4, S6, and S8. We repeated models after removing covariate health conditions, BMI and smoking risk. Results remained consistent. See Table S9 below. We repeated models after removing state fixed effects and our control for the strength of partisanship. Results remained consistent. See Table S10 below.

**Indicator Validation**

### Predictive Validity Exercises. We used the Behavioral Risk Factor Surveillance System’s 2019 county-level results to compare the measures used in this study, ‘Days of Poor Physical Health’ and ‘Days of Poor Mental Health,’ with related indicators. In Figure S3, we recorded the county-level correlations between our outcomes and the following 10 measures.

We compared days of poor physical health per month to (1) the share of residents reporting frequent physical distress (14 or more days of poor physical health), (2) those reporting poor or fair health (items 1 or 2 on a 5 point scale of self-rated health), (3) the premature death rate for adults under age 75 per 100,000 residents, (4) rates of physical inactivity in the population, and the prevalence rates of (5) diabetes and (6) obesity. We compared days of poor mental health per month to (7) the share of residents reporting frequent mental or emotional distress (14 or more days a month), (8) depression, (9) sleeping less than 7 hours a night, and (10) high blood pressure (a correlate of stress).

**Figure S3** reveals that our outcome variables are strongly correlated with these related measures, with Pearson’s correlation coefficients between 0.7 and 0.9, as well as 0.59 for obesity. Given that Pearon’s r ranges from -1 (negative association) to 0 (neutral) to +1 (positive association), these are extremely favorable results.

These demonstrate *strong predictive validity* of the types of health outcomes we would expect from someone reporting more days of poor physical or mental health.

### Conceptual Validity Exercises. After the promising results in Figure S3, as a second step, we investigated their conceptual validity using known correlates. The logic goes that if this study’s outcomes are appropriate measures of health, then they should correlate as expected with demographic traits that frequently correlate with other health indicators.

### In Figure S4, we investigate the correlations between out outcomes and five demographic correlates. These included (1) county *median income*, (2) *the share of residents with some or more college education*, and (3) *the share of unemployed residents* (these three measures were inversed for easier readability in Figure S3). We also compared (4) *the share of Black residents*, to highlight how systemic racism leads to worse health outcomes, and (5) *median age*.

For each trait, we compared the correlation with our outcome to the correlation with each of our alternative health indicators from **Figure S3.** If our outcome measures are conceptually valid, then they should demonstrate the same associations with these demographic correlates as our other health indicators.

As a simple check, we added a 0.25 width band, centered on the mean correlation for each panel in **Figure S4.** If all bars fall within this band, we regard those correlations as very close together, such that those variables correlations are an ‘excellent match’. This was the case for 4 out of our 10 panels. If just one correlation falls just slightly outside the band, we view those as still a ‘great match.’ This was the case for 4 our of our 10 panels. If just one correlation falls substantially outside of the band, we view that as a ‘fair match.’ This occurred for 2 of our panels. In these 2 cases, correlations between depression rates and the share of Black residents, as well as the median age, deviated slightly from correlations with days of poor mental health and the same demographic traits.

How should we interpret these differences? First, we should recall that days of poor mental health still correlated very strongly with depression rates overall in **Figure S3**, at r = 0.71. Second, there is a readily available explanation for why official depression rates are lower in communities of color and older counties: mental health concerns are not always spoken about as readily in these communities, and so we would expect that depression rates may be underreported due to social desirability bias, compared to other measures of poor mental health. In fact, these results in **Figure S4** suggest to the authors that days of poor mental health may be a useful metric *instead* of depression rates, since it appears to closely correlate with other measures, such as frequent mental distress, sleep deprivation, and high blood pressure (used to represent stress levels), using softer language perhaps more acceptable to a broader audience. In summary, these outcomes demonstrate *fairly strong conceptual validity* for almost all health outcomes considered, with understandably small levels of variation.

**Survey Questions**

**Description of Survey Questions.** Below, we summarize the questions used in this study that were asked in the December 2019 Qualtrics quota survey of US residents. Formatting is consolidated for ease of reading, but question wording remains the same.

**Health Questions:** See questions about health below.

Q1. Thinking about your physical health, which includes physical illness and injury, for how many days (0-30 days) during the past 30 days was your physical health not good? Source: Behavioral Risk Factor Surveillance System 2016.

Q2. Now thinking about your mental health, which includes stress, depression, and problems with emotions, for how many days (0-30 days) during the past 30 days was your mental health not good? Source: Behavioral Risk Factor Surveillance System 2016

Q3. Do you have any kind of health care coverage, including health insurance, prepaid plans such as HMOs, or government plans such as Medicare? Answers: 1) Yes, 2) No, 3) Don’t know/Not sure. Source: Behavioral Risk Factor Surveillance System 2018

Q4. Have you smoked at least 100 cigarettes in your entire life? Answers: 1) Yes, 2) No, 3) Don’t know/Not sure. Source: Behavioral Risk Factor Surveillance System 2016

Q5. Do you now smoke cigarettes every day, some days, or not at all? Answers: 1) every day, 2) some days, 3) not at all, 4) Don’t know/Not sure. Source: Behavioral Risk Factor Surveillance System 2016

Q6. About how tall are you without shoes? (in feet and inches). Source: Behavioral Risk Factor Surveillance System 2018

Q7. About how much do you weigh without shoes? (in pounds). Source: Behavioral Risk Factor Surveillance System 2018.

**Polarization Questions:** See questions about polarization below.

Q8. Next, we would like to ask you a few questions about today's political climate. The next several questions ask you to place yourself or other groups on a scale, where 0 equals most liberal, 10 equals most conservative, and 5 equals neither liberal nor conservative. Please use the scale below as a reference.


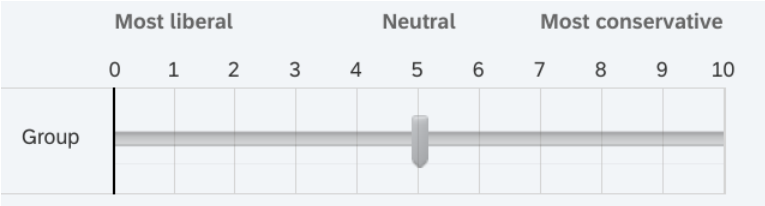


Using the scale above, where 0 equals most liberal, 10 equals most conservative, and 5 equals neither liberal nor conservative, where would you place the following now? (Answers: 0-10). Categories: yourself, the average voter in your state, the average voter in the United States as a whole.

**Politics Questions:** See questions about politics below.

Q9a. Generally speaking, do you think of yourself as a ...? Answers: 1) Democrat? 2) Republican? 3) Independent, 4) Other, 5) Not Sure (answers 1-2 randomized).

Q9b. If Democrat is Selected: Do you consider yourself to be a… Answers: 1) Strong Democrat, 2) Not very strong Democrat, 3) Not sure (answer 1-2 randomized).

Q9c. If Republican is Selected: Do you consider yourself to be a… Answers: 1) Strong Republican, 2) Not very strong Republican, 3) Not sure (answer 1-2 randomized).

Q9d. If Independent/Other/Not Sure is Selected: Do you lean towards the: Answers: 1) Democratic Party, 2) Republican Party, 3) Neither (answers 1-2 randomized). Source: Wording from YouGov Cooperative Congressional Election Survey 2016 question "pid3".

**Demographics Questions:** see questions about respondent demographics below.

Q10. What is your gender? Answer: 1) Male, 2) Female, 3) Don’t know/Not sure (answers 1-2 randomized). Source: Behavioral Risk Factor Surveillance System 2018.

Q11. Are you currently.... Answer: 1) Employed for wages, 2) Self-employed, 3) Out of work for 1 year or more, 4) Out of work for less than 1 year, 5) A homemaker, 6) A student, 7) Retired, 8) Unable to work. Source: Behavioral Risk Factor Surveillance System 2018.

Q12. Are you… Answers: 1) Married, 2) Divorced, 3) Widowed, 4) Separated, 5) Never married, 6) a member of an unmarried couple (answers 1-6 randomized). Source: Behavioral Risk Factor Surveillance System 2018.

Q13. In what year were you born? Source: Wording from YouGov Cooperative Congressional Election Survey 2016 question "birthyr".

Q14. What is the highest grade or year of school you completed? Answers: 1) Never attended school or only kindergarten, 2) Grades 1 through 8 (Elementary), 3) Grades 9 through 11 (Some high school), 4) Grade 12 or GED (High school graduate), 5) College 1 year to 3 years (Some college or technical school), 6) College 4 years or more (College graduate). Source: Behavioral Risk Factor Surveillance System 2018

Q15. Thinking back over the last year, what was your family's annual income? Answers: 1) Less than $10,000, 2) $10,000 - $19,999, 3) $20,000 - $29,999, 4) $30,000 - $49,999, 5) $50,000 - $69,999, 6) $70,000 - $99,999, 7) $100,000 - $124,999, 8) $125,000 - $149,999, 9) $150,000 - $199,999, 10) $200,000 - $249,999, 11) $250,000 or more, 12) Prefer not to say.

Note: Race/Ethnicity variable provided by Qualtrics, used in their quota sampling process. Categories include: (Black, White, Asian, Hispanic, Other Race).


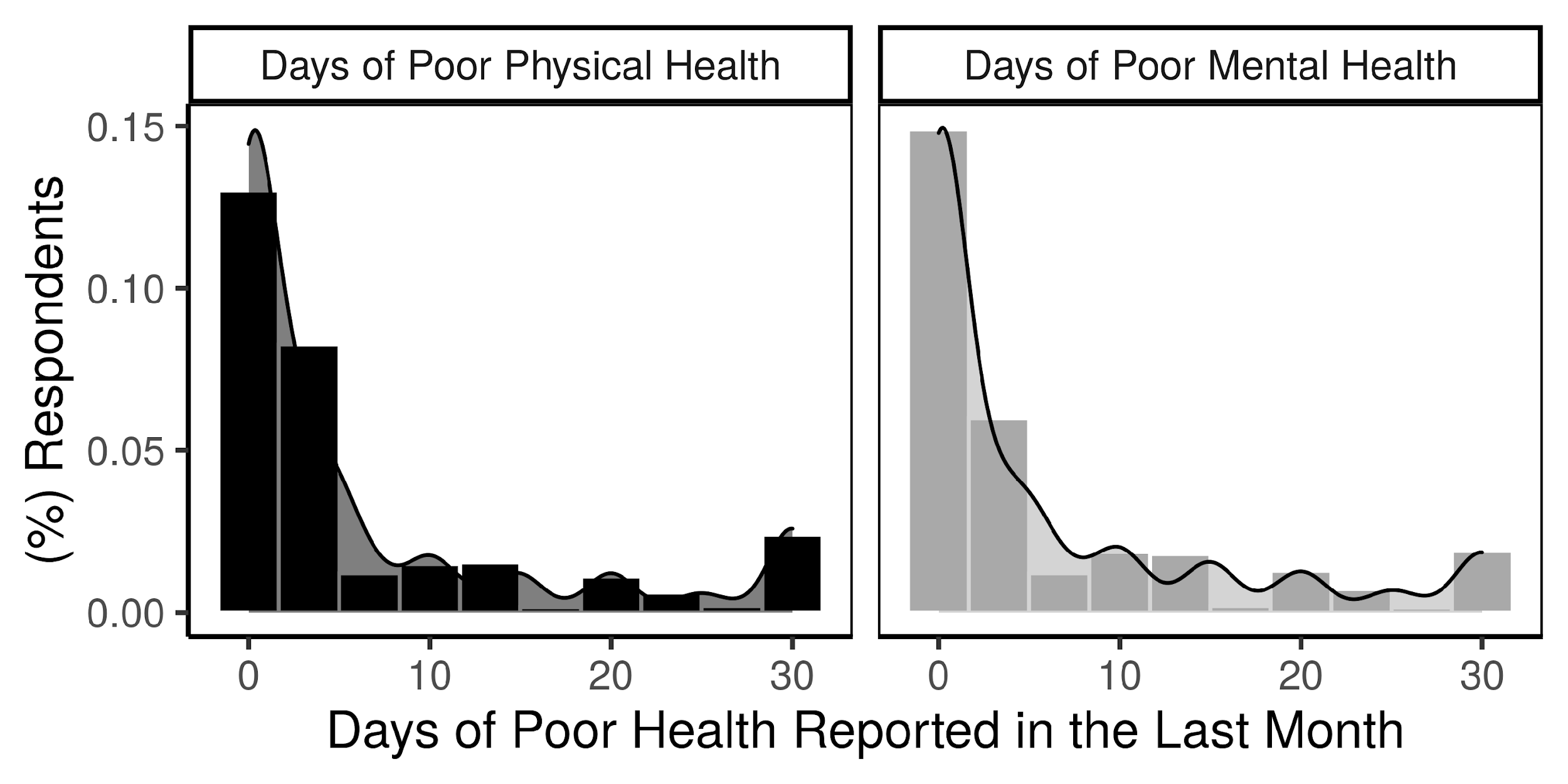


### Figure S1. Distribution of Outcome Variables from Polarization & Health Survey


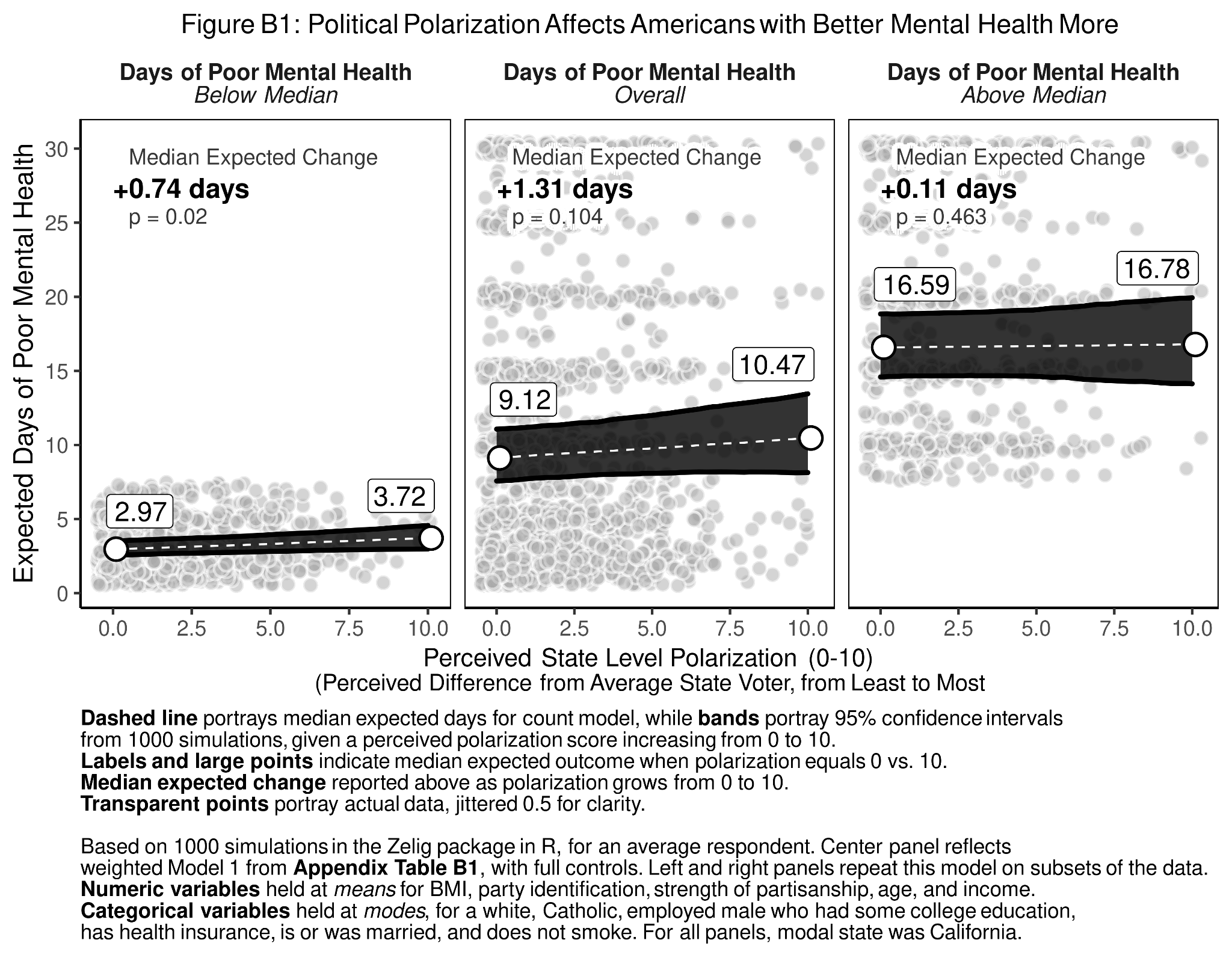


###

### Figure S2. Political Polarization Affects Americans with Better Mental Health More. *(Dashed line portrays median expected days for count model, while bands portray 95% confidence intervals from 1000 simulations, given a perceived polarization score increasing from 0 to 10. Labels and large points indicate median expected outcome when polarization equals 0 vs. 10. Median expected change reported above as polarization grows from 0 to 10. Transparent points portray actual data, jittered 0.5 for clarity. Based on 1000 simulations in the Zelig package in R, for an average respondent. Center panel reflects weighted Model 1 from Table S3, with full controls. Left and right panels repeat this model on subsets of the data. Numeric variables held at means for BMI, party identification, strength of partisanship, age, and income. Categorical variables held at modes, for a white, Catholic, employed male who had some college education, has health insurance, is or was married, and does not smoke. For all panels, modal state was California.)*

###

**
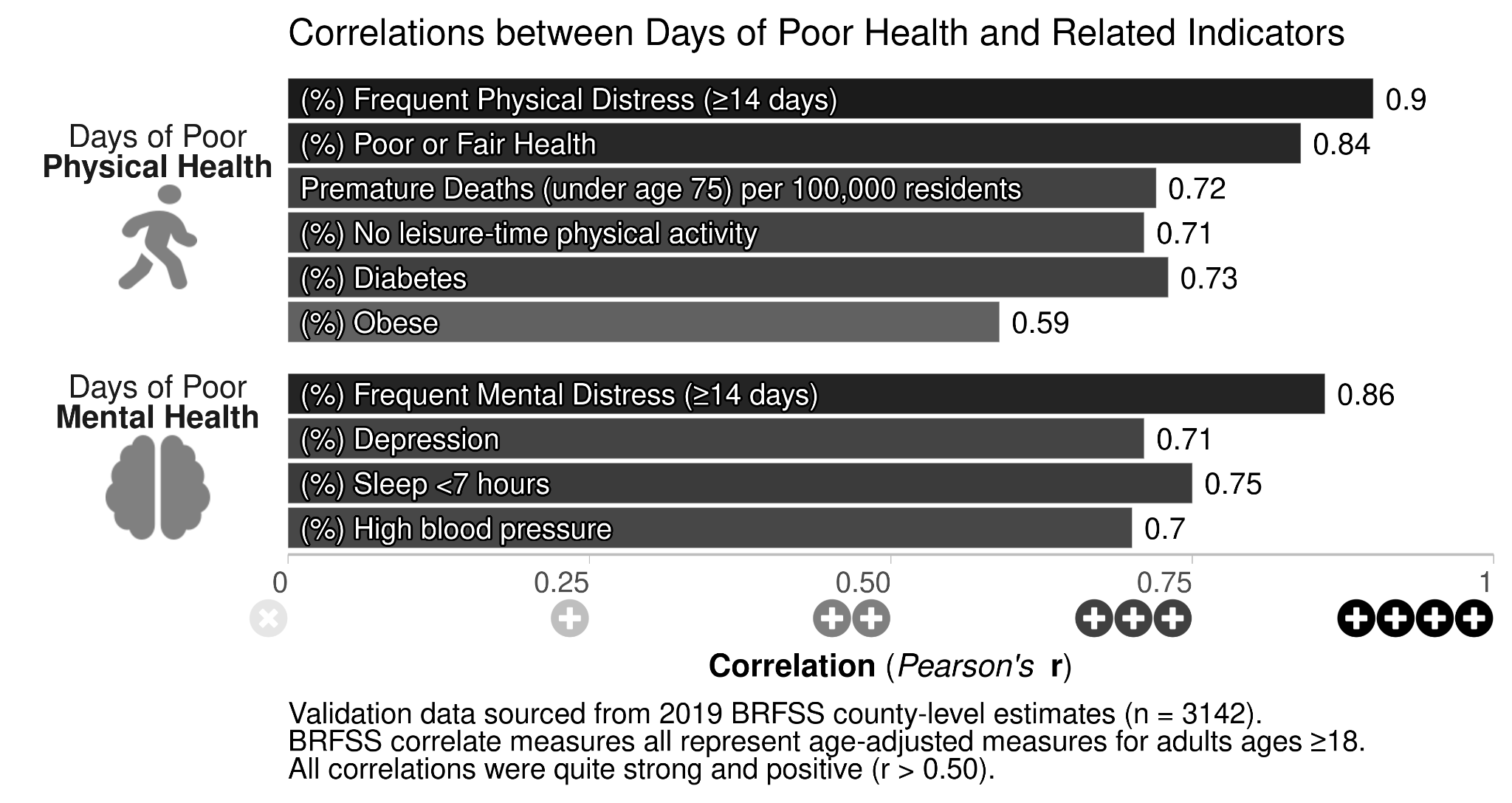
**

**Figure S3.** Associations between Health Indicators. (Validation data sources from 2019 BRFSS county-level estimates (n = 3142). BRFSS correlates represent age-adjusted measures for adults ages ≥18. All correlations were quite strong and positive (r > 0.50).)

**
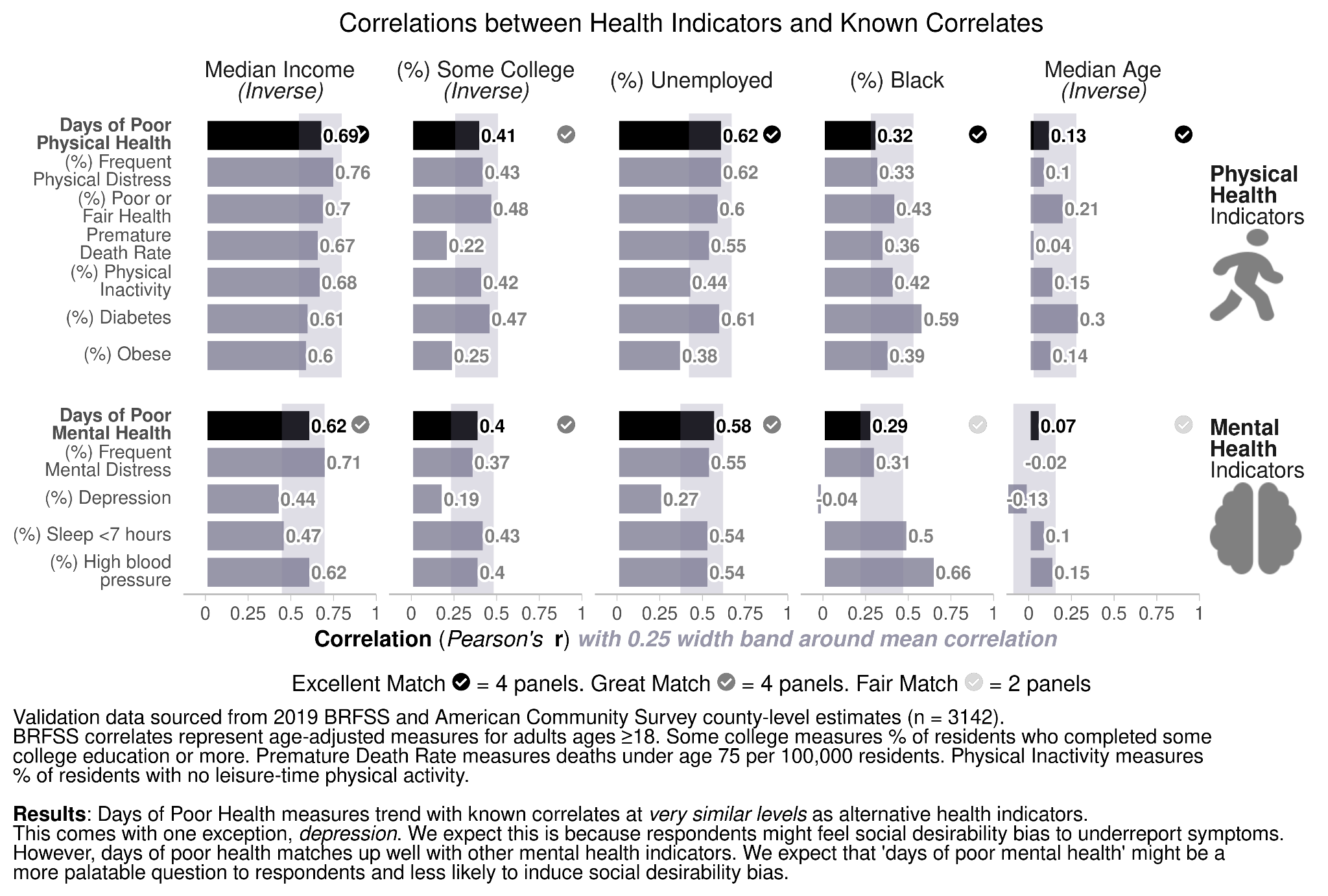
**

### Figure S4. Associations with Known Demographic Correlates of Health. *(Validation data sourced from 2019 BRFSS and American Community Survey county-level estimates (n = 3142). BRFSS correlates represent age-adjusted measures for adults ages ≥18. Some college measures % of residents who completed some college education or more. Premature Death Rate measures deaths under age 75 per 100,000 residents. Physical Inactivity measures % of residents with no leisure-time physical activity. Results: Days of Poor Health measures trend with known correlates at very similar levels as alternative health indicators. This comes with one exception, depression. We expect this is because respondents might feel social desirability bias to underreport symptoms. However, days of poor health matches up well with other mental health indicators. We expect that ‘days of poor mental health’ might be a more palatable question to respondents and less likely to induce social desirability bias.)*

###
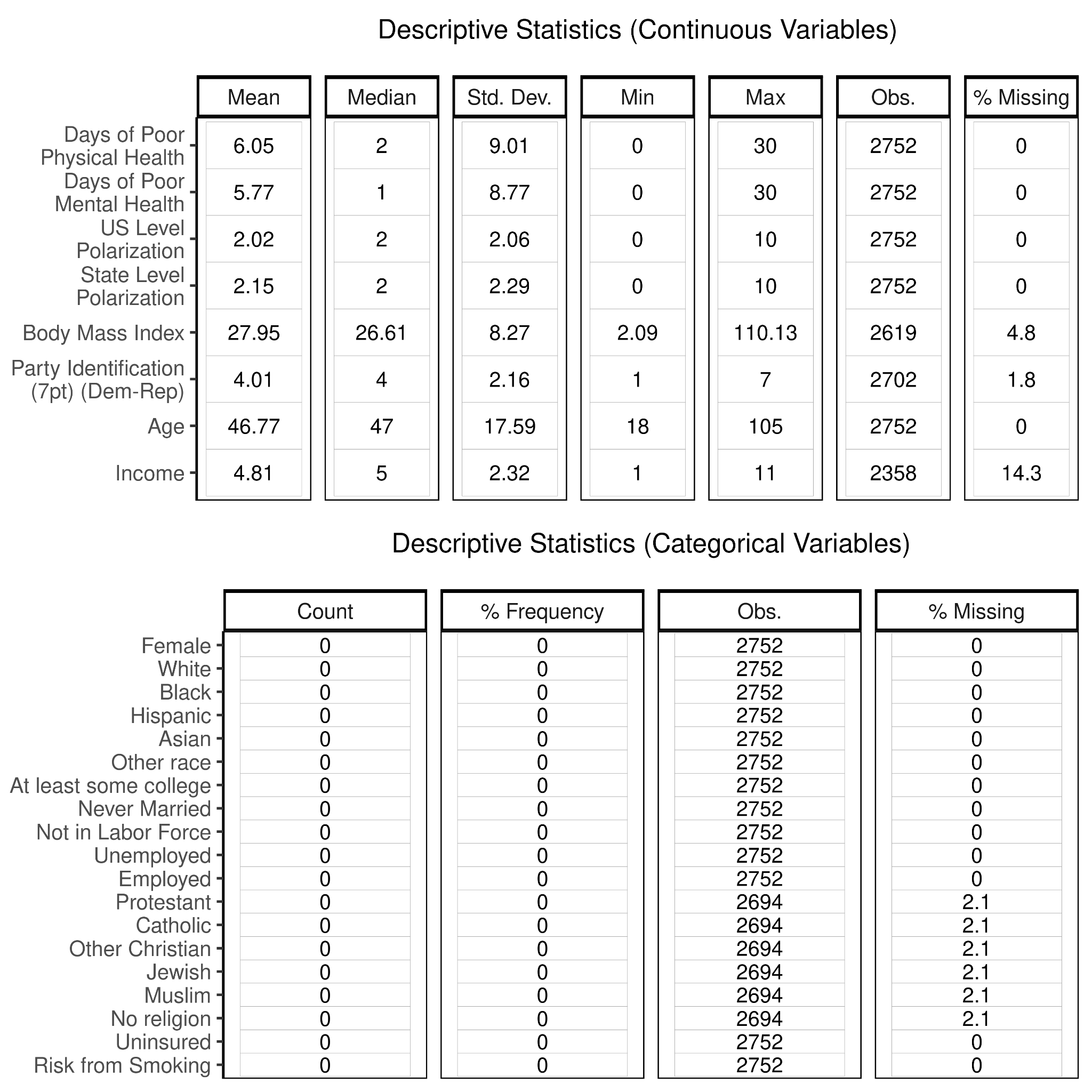


### Table S1. Descriptive Statistics for Weighted Variables


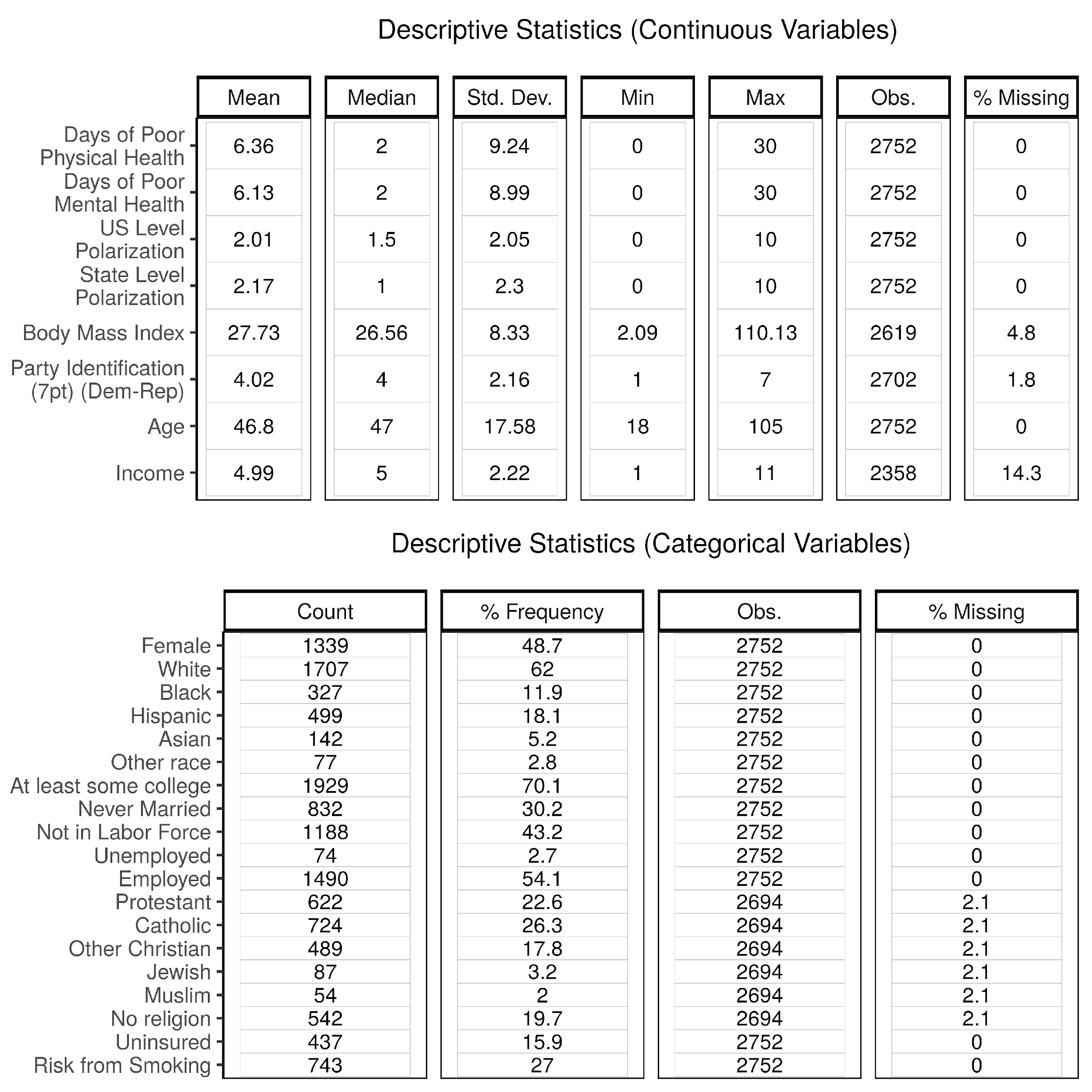


### Table S2. Descriptive Statistics for Unweighted Variables


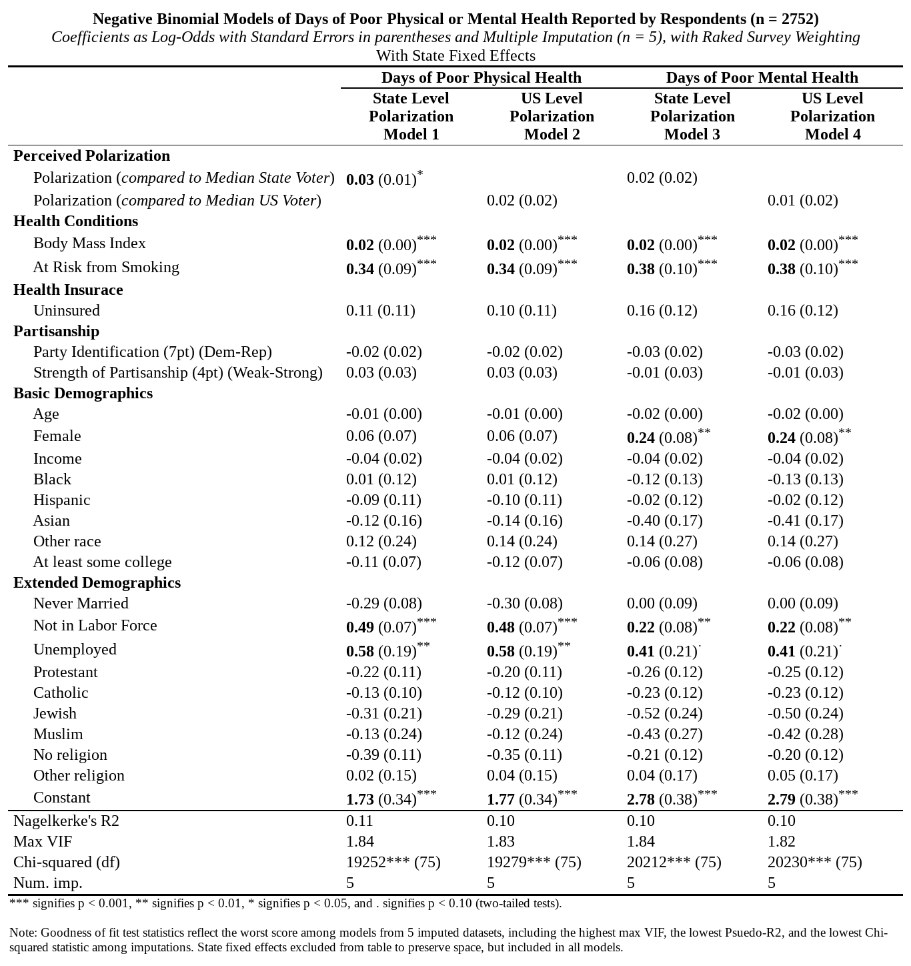


### Table S3. Negative Binomial Models of Effects of Political Polarization (Weighted Models 1-4)

####


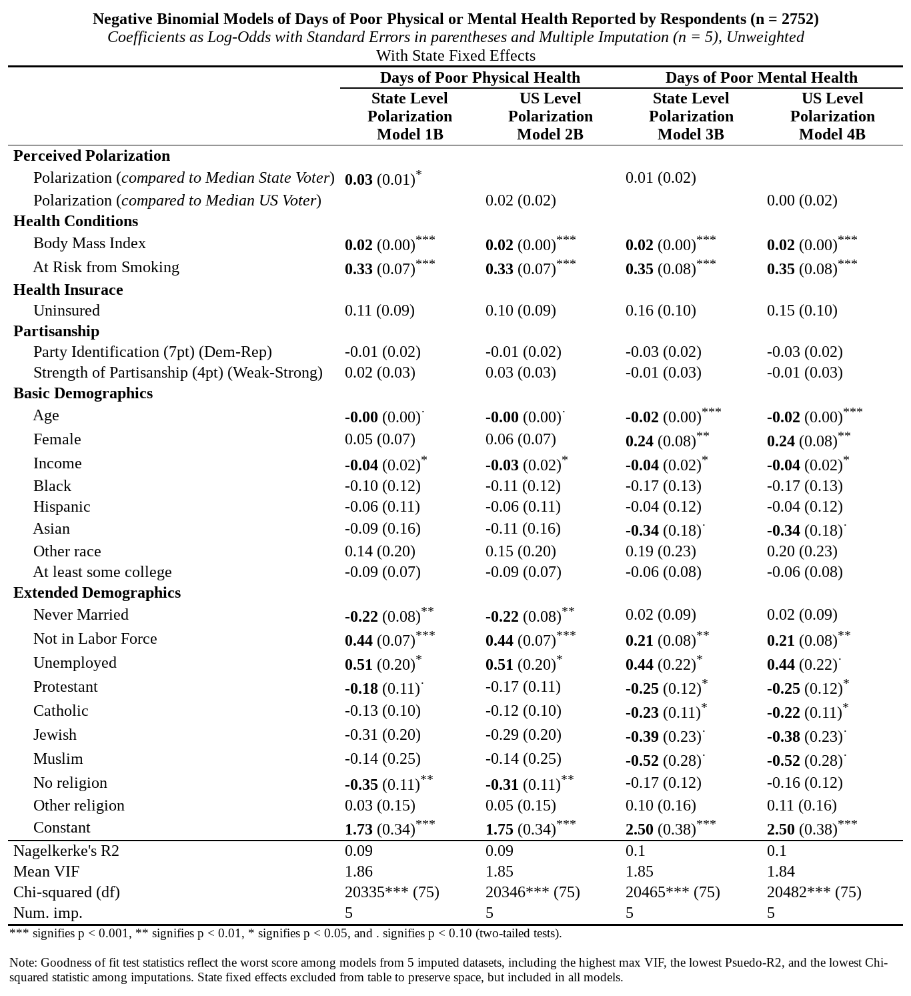


**Table S4**. Negative Binomial Models of Effects of Political Polarization (Unweighted Models 1B-4B)

**
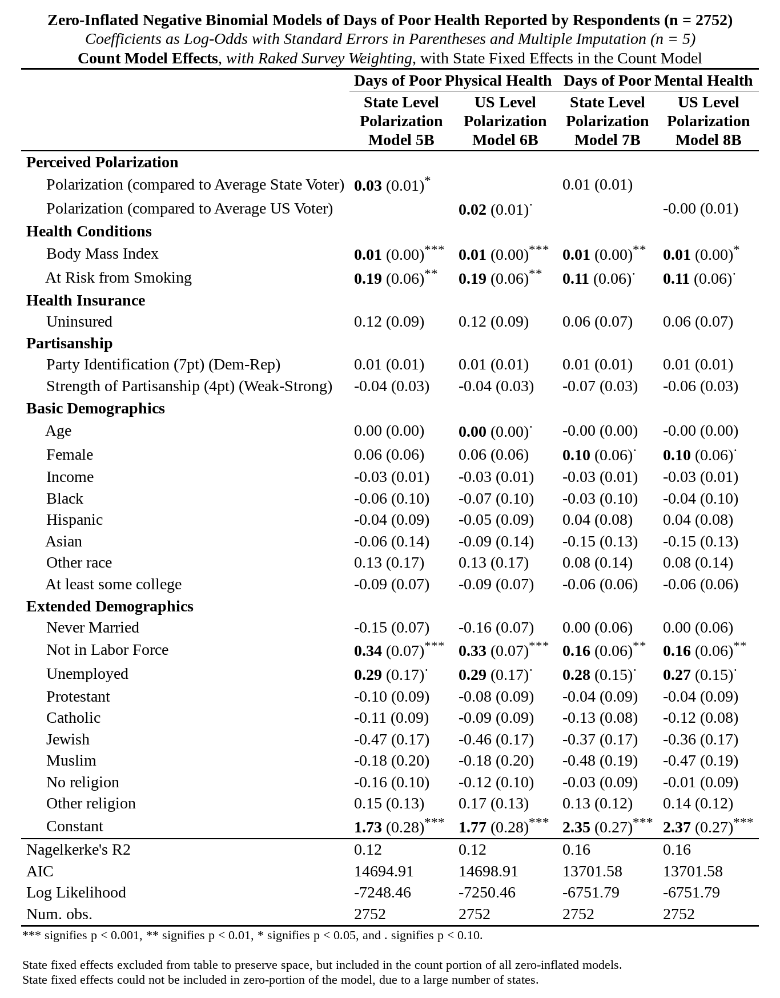
**

**Table S5.** Zero-Inflated Negative Binomial Count Effects of Political Polarization (Weighted Models 5-8; Count portion)


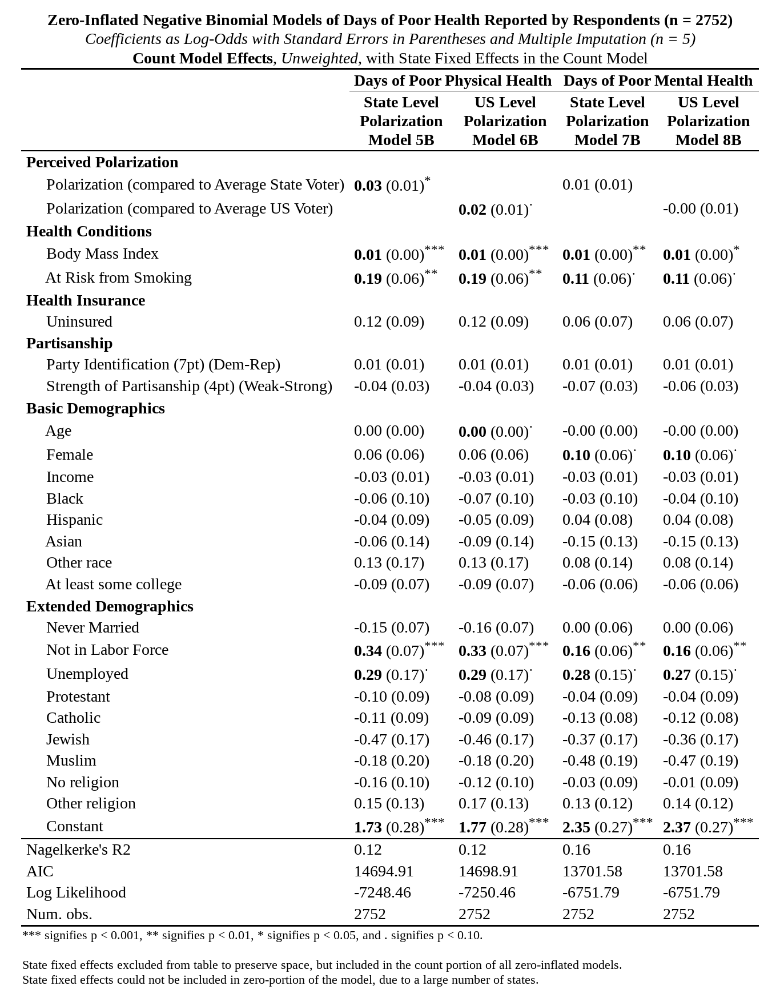


**Table S6.** Zero-Inflated Negative Binomial Count Effects of Political Polarization (Unweighted Models 5B-8B; Count portion)

**
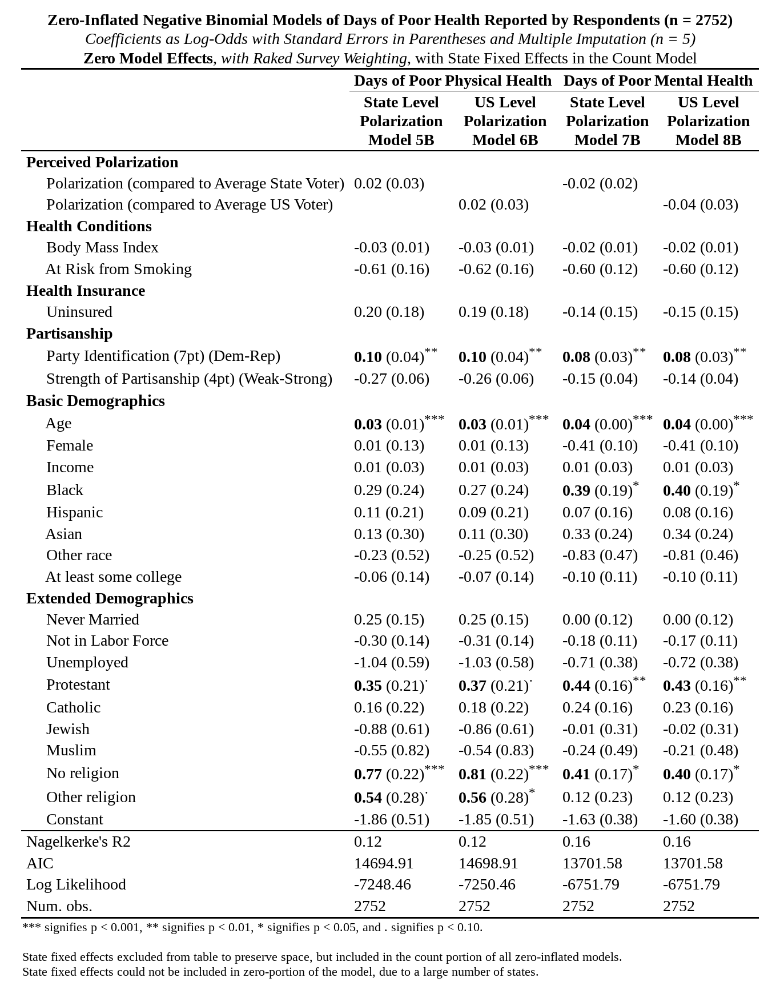
**

### Table S7. Zero-Inflated Negative Binomial Zero Effects of Political Polarization (Weighted Models 5-8; Zero portion)

####


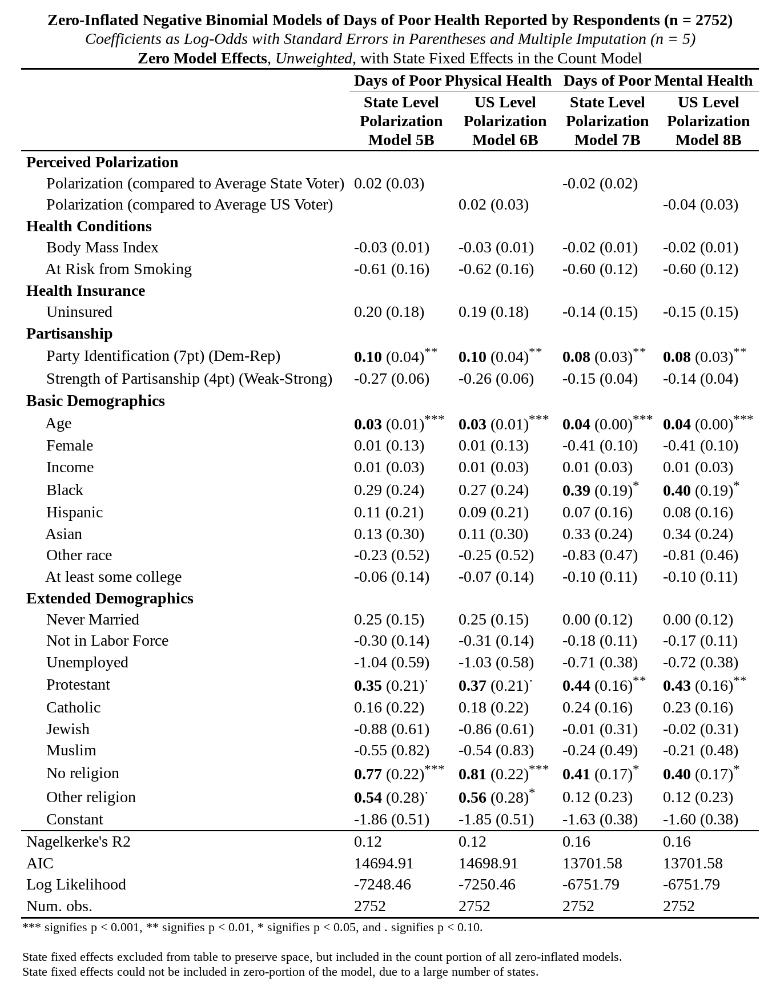


**Table S8.** Zero-Inflated Negative Binomial Zero Effects of Political Polarization (Unweighted Models 5B-8B; Zero portion)

###
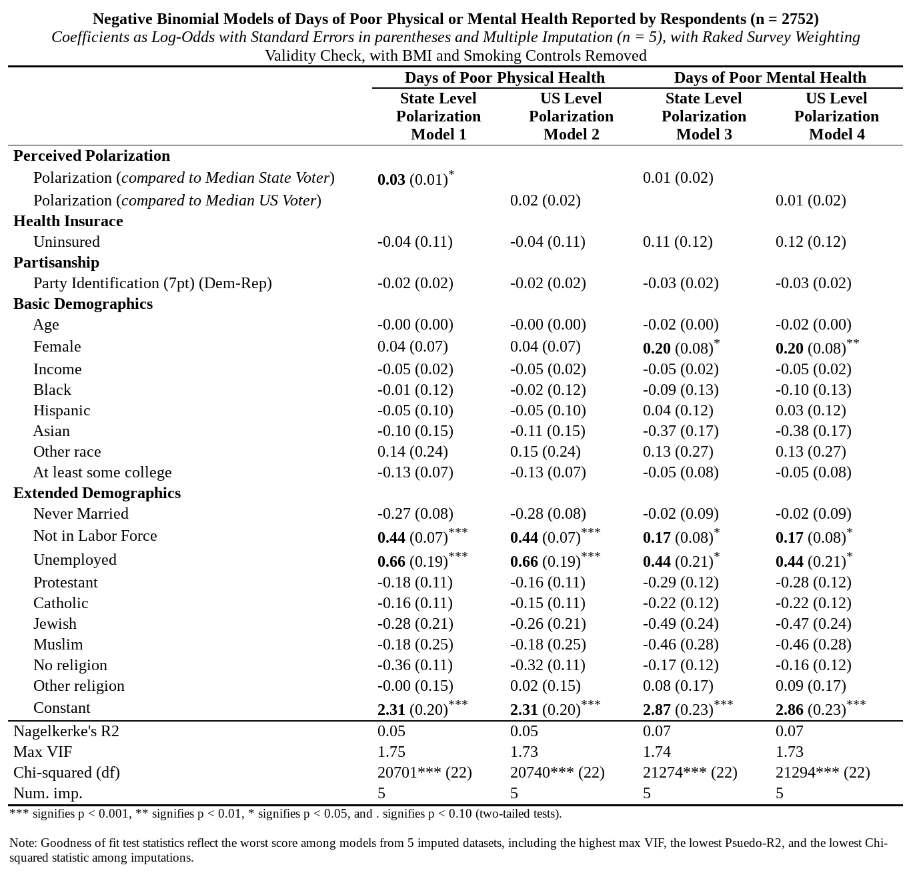


**Table S9.** Models remain consistent with BMI, Smoking, and State Fixed Effects Removed (see Table S3 for comparison with original)

###
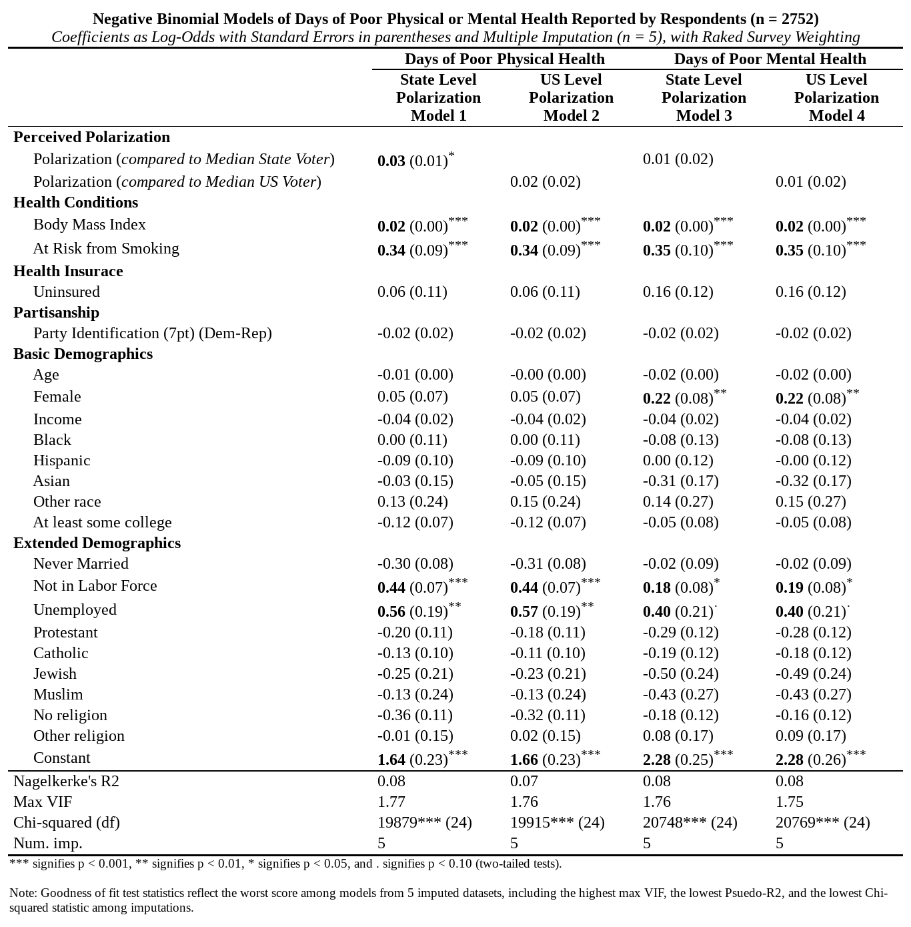


### Table S10. Models remain consistent with Strength of Partisanship and State Fixed Effects Removed (see Table S3 for comparison with original)

**SI References**

1. T. Lumley. Survey Package: Analysis of Complex Survey Samples. *CRAN*. <http://r-survey.r-forge.r-project.org/survey/> (2020).
2. F. A. Potter. Study of Procedures to Identify and Trim Extreme Sample Weights. *Proceedings of the Survey Research Methods Section, American Statistical Association*, pp. 225-230 (1990).
